# Protein target similarity is positive predictor of in vitro antipathogenic activity: a drug repurposing strategy for *Plasmodium falciparum*

**DOI:** 10.1186/s13321-024-00856-7

**Published:** 2024-05-30

**Authors:** Reagan M. Mogire, Silviane A. Miruka, Dennis W. Juma, Case W. McNamara, Ben Andagalu, Jeremy N. Burrows, Elodie Chenu, James Duffy, Bernhards R. Ogutu, Hoseah M. Akala

**Affiliations:** 1 Center for Research On Genomics and Global Health, National Human Genome Research Institute, National Institutes of Health, Bethesda, MD USA; 2 Center for Clinical Research, Kenya Medical Research Institute (KEMRI), P. O. Box 54, Kisumu, 40100 Kenya; 3 Center for Research in Therapeutic Sciences, Strathmore University, P.O. Box 59857-00200, Nairobi, Kenya; 4 Calibr-Skaggs Institute for Innovative Medicine, a division of The Scripps Research Institute, La Jolla, CA USA; 5 Medicines for Malaria Venture, Geneva, Switzerland; 6 Department of Emerging Infections Diseases (DEID), Walter Reed Army Institute of Research – Africa, Kisumu, Kenya

**Keywords:** Drug repurposing, Drug discovery, Drug development, Computer aided drug discovery, ReFRAME, Antimalarial, Antiplasmodial, Mutagenesis fitness score, Mutagenesis index score

## Abstract

Drug discovery is an intricate and costly process. Repurposing existing drugs and active compounds offers a viable pathway to develop new therapies for various diseases. By leveraging publicly available biomedical information, it is possible to predict compounds’ activity and identify their potential targets across diverse organisms. In this study, we aimed to assess the antiplasmodial activity of compounds from the Repurposing, Focused Rescue, and Accelerated Medchem (ReFRAME) library using in vitro and bioinformatics approaches. We assessed the in vitro antiplasmodial activity of the compounds using blood-stage and liver-stage drug susceptibility assays. We used protein sequences of known targets of the ReFRAME compounds with high antiplasmodial activity (EC_50_ < 10 uM) to conduct a protein-pairwise search to identify similar *Plasmodium falciparum* 3D7 proteins (from PlasmoDB) using NCBI protein BLAST. We further assessed the association between the compounds' in vitro antiplasmodial activity and level of similarity between their known and predicted *P. falciparum* target proteins using simple linear regression analyses. BLAST analyses revealed 735 *P. falciparum* proteins that were similar to the 226 known protein targets associated with the ReFRAME compounds. Antiplasmodial activity of the compounds was positively associated with the degree of similarity between the compounds’ known targets and predicted *P. falciparum* protein targets (percentage identity, E value, and bit score), the number of the predicted *P. falciparum* targets, and their respective mutagenesis index and fitness scores (R^2^ between 0.066 and 0.92, *P* < 0.05). Compounds predicted to target essential *P. falciparum* proteins or those with a druggability index of 1 showed the highest antiplasmodial activity.

**Supplementary Information:**

The online version contains supplementary material available at 10.1186/s13321-024-00856-7.

## Scientific contribution

This is the first study to demonstrate a correlation between in vitro antipathogenic activity of compounds and target similarity across species. Our findings indicate that leveraging protein-target similarity may accelerate the drug repurposing process for many diseases by predicting compounds’ activity and their prospective targets in different organisms.

**Supplementary Information:**

The online version contains supplementary material available at 10.1186/s13321-024-00856-7.

## Introduction

The process of drug discovery and development is long, costly, and complex, involving various stages of preclinical and clinical testing before a new drug can be approved for use [1]. However, repurposing known drugs for new indications has emerged as an alternative approach to traditional drug development, given its potential for reducing the time and cost involved in bringing a drug to market.

Drug repurposing represents a more rapid and cost-effective pathway, with reduced risks compared to conventional drug discovery methods [2]. Traditional approaches suffer from high attrition rates, with many promising compounds failing approval due to safety and effectiveness concerns [3]. In contrast, drug repurposing leverages publicly available biomedical data, knowledge of human safety and tolerability and harnessing the potential of approved or investigational drugs to uncover novel applications or to enhance the potency of existing solutions [2].

Repurposing is challenging, however, since selective efficacy is required in a drug. Since drugs are optimised for a specific target and indication, a successful repurposing exercise requires the new activity to be even more potent on a new target, or for the inhibition of the original target to lead to new benefits, with acceptable safety and tolerability, in a new indication [2]. Thus, the success of repurposing in delivering new therapies is limited, though at the same time significant new biological insights can be obtained.

Repurposing opportunities can be achieved by identifying known drugs' potential targets in different diseases or organisms through a protein similarity approach. Knowing the potential protein targets of therapeutic agents serves as a crucial tool for discovering and optimizing active compounds [4]. An effective drug against a pathogen should interact with a protein critical to the pathogen's survival or transmission. These protein targets would be regarded as essential and would have high druggability indices [5].The similarity between analogous protein targets can be used to predict compounds with activity, as seen in cases such as *Plasmodium falciparum* [6] and *Schistosoma mansoni* [7]. Moreover, open data sources are enriching this field, providing essential insights into proteins, approved drugs, essentiality of proteins, druggability and potential biochemical pathways that may be exploited in drug repurposing [8].

In the context of malaria, one of the deadliest infectious diseases [9], repurposing existing drugs for the treatment of malaria has the potential to significantly reduce the burden of the disease, especially in resource-limited settings. *P. falciparum* is the most virulent of the five *Plasmodium* species that cause malaria in humans and is responsible for most malaria-related deaths [9]. As the resistance to existing antimalarials continues to grow [10], the development of new, effective antimalarial drugs is becoming increasingly urgent to maintain progress in controlling and eliminating malaria worldwide [10]. Therefore, identifying compounds with activity against *P. falciparum* is a critical step in developing effective treatments for malaria.

The urgent need to discover and develop therapeutics against a wide array of pathogens necessitates the identification of novel active compounds and the elucidation of their molecular targets. Target similarity can be used in predicting compounds with activity and identifying their potential targets. Our study aimed to predict the targets of compounds with antiplasmodial activity and explore the association between the compounds’ activity and the similarity between their known and predicted *P. falciparum* targets*.* To our knowledge, this approach marks the first of its kind in analyzing such target associations in any species, contributing a new perspective to drug repurposing. We utilized the Repurposing, Focused Rescue, and Accelerated Medchem (ReFRAME) library for our analyses since it comprises approximately 12,000 curated compounds, each of which has been subjected to extensive clinical development or thorough preclinical profiling [11]. Our methodology included drug susceptibility assays to evaluate the in vitro activity of the compounds, and database searches aimed at identifying *P. falciparum* protein targets similar to those known for the ReFRAME compounds. We utilized NCBI's protein BLAST [12] and the Consurf server [13] to facilitate these protein similarity analyses, and the Tropical Disease Research (TDR) database to derive essentiality and druggability indices for the predicted *P. falciparum* protein targets. We evaluated the association between the compounds' in vitro antiplasmodial activity with the sequence similarity of the known and predicted target pairs, and the essentiality and druggability of the predicted *P. falciparum* targets. Our findings could provide a foundation for developing new anti-parasitic therapies.

## Methods

### Laboratory assays

In vitro drug susceptibility assays and toxicity assays were conducted at Calibr at Scripps Research, La Jolla, CA, USA, according to the procedures detailed below.

### *P. falciparum* cultures

The *P. falciparum* Dd2-luciferase-expressing line (Dd2-HLH; a gift from Prof. David A. Fidock (Columbia University)) was maintained using standard culturing [14] an atmosphere of 93% N_2_, 4% CO_2_, 3% O_2_ at 37 °C in complete culturing medium (10.4 g/L RPMI 1640 (without phenol red, with 2.1 mM glutamine), 5.94 g/L HEPES, 5 g/L Albumax II, 50 mg/L hypoxanthine, 2.1 g/L sodium bicarbonate, 10% human serum and 43 mg/L gentamicin). Human erythrocytes served as the host cell to support propagation with a final hematocrit of 2.5%.

### *P. falciparum* asexual blood stage assays

In vitro antimalarial activity was independently measured using three independent assays: a 72 h SYBR Green proliferation assay, and a luciferase-based viability assay that either read out at 48 h or 96 h to distinguish between standard-acting and slow-acting compounds, respectively. The SYBR Green cell proliferation assay followed a previously described method for screening in 1,536-well format (SYBR assay [15]). Likewise, the luciferase-based viability assay followed the same protocol referenced for the SYBR assay, except that luminescence was measured at the end of the assay (either a 48 or 96-h incubation) using a and 2 µL/well dispense of Bright-Glo™ Luciferase Assay System reagent (Promega). Plates were then read for 1 s on a Viewlux luminescence reader.

### *P. berghei* liver-stage viability assay

Liver-stage activity of reconfirmed hits from the ReFRAME screen were determined using an in vitro assay established by Meister et al., [16]. In brief, a HepG2 cell line was used to support the complete development of rodent-malaria sporozoites [17]. A continuous in vitro culture of this cell line was maintained at 37 °C in 4% CO_2_ in complete media containing DMEM (Invitrogen) supplemented with 10% fetal calf serum, 0.29 mg/ml glutamine, 100 units penicillin and 100 μg/ml streptomycin (Sigma-Aldrich). One day prior to sporozoite infection, a MultiFlo dispenser (Biotek; 1 µl cassette) was used to seed 3,000 HepG2 cells/well into a white, solid 1536-well microtiter plate (Greiner). Plates were incubated at 37 °C in 4% CO_2_ overnight. The following day, 10 nL of DMSO-dissolved compounds/well were acoustically transferred (Labcyte Echo) to the microtiter assay plate. The DMSO concentration did not exceed 0.1% and 1 µM atovaquone (final concentration) was used as a positive control for normalization. *Anopheles stephensi* mosquitoes infected with *P. berghei*-luciferase (provided by New York University Langone Medical Center Insectary), were dissected to recover sporozoites from the mosquito salivary glands. An automated dispense (BioTek MultiFlo; 1 µL cassette) was used to deliver 750 sporozoites/well. The final assay volume was 10 µL and plates were incubated at 37 °C at 4% CO_2_ for 48 h. Parasite viability was detected by dispensing BrightGlo (Promega) and luminescence was immediately measured with an EnVision (PerkinElmer).

### Mammalian cell cytotoxicity assays

Two mammalian cell lines were used for counter-screening for general cytotoxicity of hit compounds: human embryonic kidney cells (HEK293T; ATCC CRL-3216) and human hepatocellular carcinoma cells (HepG2; ATCC HB-8065). Each were maintained in T-150 tissue culture flasks with DMEM supplemented with 10% HI-FBS, 100 IU penicillin, and 100 mg/mL streptomycin. At 80% confluency, cells were trypsinized, washed, and resuspended in assay medium: DMEM supplemented with 2% HI-FBS, 100 IU penicillin, and 100 mg/mL streptomycin. Compounds were pre-spotted into tissue culture-treated white solid-bottomed 1536-well plates (Greiner) in a 1:3 dose–response dilution (top concentration 20 μM). HEK293T and HepG2 cells were diluted to 75 × 10^3^ cells/mL and 150 × 10^3^ cells/mL, respectively, and 5 μL/well were dispensed into assay plates with a MultiFlo FX Multi-Mode Dispenser (Biotek). Cells were incubated with metal lids (The Genomics Institute of the Novartis Research Foundation) at 37 °C with 5% CO_2_ in a humidified tissue culture incubator for 72 h. At the completion of the assay, CellTiter-Glo (Promega) was prepared at 1:2 (reagent:water) of the manufacturer’s instructions, and 2 μL were dispensed into each well. After a 5-min incubation at room temperature, luminescence readings were measured with an EnVision Multilabel Plate Reader (Perkin Elmer). Relative fluorescence units were uploaded into Genedata Screener (v13.0-Standard), and data normalized to DMSO- and puromycin-treated wells. A four-parameter non-linear regression curve fit was applied to dose–response data using Genedata to determine the half-maximal cytotoxic concentration (CC_50_) of each compound.

### ReFRAME screening workflow

Three independent, primary screens of the ReFRAME library were carried out against *P. falciparum* Dd2-HLH at a final screen concentration of 1.25 µM. Primary screen hits were defined as those wells generating ≥ 50% inhibition in fluorescence or luminescence signal compared to inhibitor (10 µM artemisinin) minus control (DMSO) well normalization. Primary hits were directly advanced into concentration–response curves using a 12-point, 1:3 dilution series with a top concentration of 12.5 µM. Data were fit with Genedata Analyzer using the Smart Fit function. Final filtered hits included those with an EC_50_ (half-maximal effective concentration) ≤ 10 µM. For additional evaluation, cytotoxicity against human cell lines (CC_50;_ half-maximal cytotoxic concentration) was provided to inform on compound selectivity with a SI ≥ 10 being ideal (SI = CC_50_ / EC_50_) for both cell lines. Final data are available at https://reframedb.org, an open access resource supported by Calibr-Skaggs and the Bill & Melinda Gates Foundation.

### Protein target similarity analyses

The aim of similarity analyses was to identify potential targets among *P. falciparum* proteins for active ReFRAME compounds. We selected ReFRAME compounds that exhibit high antiplasmodial activity (EC_50_ < 10 µM) and are not currently used as antimalarials. Using their known protein targets, we searched for similar *P. falciparum* proteins, based on the hypothesis that a compound is more likely to target a *P. falciparum* protein if there is a structural resemblance to its established target. Searches for known drug targets were conducted on Google Scholar (https://scholar.google.com), PubMed (https://pubmed.ncbi.nlm.nih.gov/), DrugBank (https://go.drugbank.com/), and the Therapeutic Target Database (http://db.idrblab.net/) using the names of the ReFRAME compounds under default settings. Protein sequences of the known drug targets were retrieved from UniProt using their accession numbers, and the complete *P. falciparum* (strain 3D7) proteome was obtained from PlasmoDB (version 47, https://plasmodb.org/). We created a local protein database of *P. falciparum 3D7* proteome from the fasta file downloaded from PlasmoDB (4.PlasmoDB-47_Pfalciparum3D7_AnnotatedProteins.fasta) using the command ‘*makeblastdb -in 4.PlasmoDB-47_Pfalciparum3D7_AnnotatedProteins.fasta -dbtype prot’*. We used the known target sequences (listed in ‘query_seqs.fasta’) to query a local database of the *P. falciparum* proteome using the Basic Local Alignment Search Tool (BLAST version 2.12.0, https://blast.ncbi.nlm.nih.gov/) [12] using ‘*blastp -query query_seqs.fasta -db 4.PlasmoDB-47_Pfalciparum3D7_AnnotatedProteins -out orthologs.txt -outfmt 6.*’. The output was formatted in a tab-delimited text file (*orthologs.txt*), which was used to identify potential orthologs. Known target and *P. falciparum* protein pairs with alignment with greater than 30% similarity were considered for further analyses, as this level of similarity is suggested to have sufficient similarity for analogous proteins [18].

In local BLAST analyses, multiple outputs may be generated for each protein-pairwise alignment. This feature indicates the tool's sensitivity in detecting and reporting various regions within the protein sequences that align significantly with the query sequence. Each output represents a specific segment of alignment, reflecting the ability to identify regions of similarity that may differ in biological functions or structural characteristics. Key metrics for each alignment include the E-value, percentage identity, and bit score. The E-value indicates the likelihood of an alignment with a similar score occurring by chance, with lower values signifying greater significance. The percentage identity measures the proportion of identical residues in the alignment, directly indicating similarity. The bit score normalizes the raw alignment score to facilitate comparisons across different searches. This detailed output in local BLAST contrasts with the more consolidated summaries provided by online BLAST analyses, which often present an overall alignment view for each query-target pair. Such detail is particularly crucial for understanding the nuances of each protein interaction.

### Similarity of functional amino acid residues

Functional or structural amino acids in homologous proteins are conserved across species and hence are more likely to be shared in proteins that have similar functions and structures. We evaluated the percentage of conserved functional or structural amino acids shared between the known targets and their corresponding *P. falciparum* predicted targets to fine-tune the similarity analyses. We identified structural and functional amino acids in the known drug targets using the ConSurf Server with default parameters, for detailed methodologies and parameters refer to https://consurf.tau.ac.il/ [13]. We determined the percentage of functional and structural amino acids that were conserved between the known protein–predicted *P. falciparum* target pairs (Supplementary Fig. 1).

### Essentiality and druggability index of predicted *P. falciparum* targets

To determine the feasibility of the *P. falciparum* orthologs as drug targets, we retrieved their druggability and essentiality data from the Tropical Disease Research (TDR) database (https://tdrtargets.org/). For this step, we selected *P. falciparum* proteins from the most similar pair of the known and predicted targets for each compound. We performed a search query using the PlasmoDB ID using default settings. Essentiality indicates how crucial a protein is in a parasite’s survival, while druggability index, which ranges from 0.1 to 1.0, is a measure of how likely it is for an oral druglike molecule to bind to the protein and bring about a therapeutic effect [5]. *P. falciparum* proteins that are essential and druggable are more likely to be effective antiplasmodial targets than those that are dispensable with low druggability indices [5, 19]. Where specific *P. falciparum* data were lacking, we retrieved essentiality data for related organisms from the TDR database. In addition, we obtained the mutagenesis index score (MIS, an indicator of gene-mutability of a protein) and mutagenesis fitness score (MFS, a measure of the impact of a mutation of a protein on the fitness or viability of an organism or a cell) of the predicted *P. falciparum* targets from a study by Zhang et al., [20]. Essential *P. falciparum* blood-stage growth proteins typically have a low MIS and MFS [20].

### Molecular docking

We performed in silico docking simulations using PyRx software (version 0.9), virtual screening software for computational drug discovery, as previously described [21], to compare binding sites and affinities of the compounds on their known targets and predicted *P. falciparum* targets. The compounds’ 3D structures in Structure-Data File (SDF) format were obtained using Openbabel (version 2.40) (http://openbabel.org). Protein 3D structures were downloaded from the Protein Data Bank (http://www.rcsb.org/) and any missing structures were modelled using SWISS-MODEL (http://swissmodel.expasy.org/). Docking simulations using a grid box that covered the entire protein were conducted with AutoDock Vina as implemented in PyRx. Docking conformations were visualized using Pymol (http://pymol.org/).

### Association between in vitro antiplasmodial activity and similarity of known protein target–predicted *P. falciparum* target pairs

We hypothesized that compounds with known targets that more closely resemble essential *P. falciparum* proteins are likely to exhibit more potent antiplasmodial activity. To test this hypothesis, we performed simple linear regression analyses to assess the association between in vitro antiplasmodial activity (EC_50_ at 48-h and 72-h asexual blood-stage assays, and EC_50_ at 48-h liver-stage assay) of the 143 drug compounds and the similarity between the known targets and predicted *P. falciparum* targets (that is percentage protein similarity, similarity bit scores, percentage of shared structural and functional amino acids) and fitness scores (MIS and MFS). Additionally, we assessed how the number of predicted *P. falciparum* targets per known target was associated with a compound’s in vitro antiplasmodial activity, noting that many compounds have multiple known targets, each of which may have several *P. falciparum* orthologs. We log-transformed the in vitro antiplasmodial activity estimates (EC_50_), percentage similarity, and bit score to normalize their distribution in the regression models. We visualized these correlations using scatter plots and compared the average antiplasmodial activity across different essentiality categories and druggability indices using boxplots. We used R (version 3.5.1) for statistical analyses and plotting of graphs. The scripts and datasets supporting the analyses of this study are accessible on GitHub in the 'Similarity_Target_Prediction' repository at https://github.com/rmogire/Similarity_Target_Prediction. This repository contains detailed documentation on the use and purpose of each code, as well as metadata for all datasets, enhancing reproducibility and facilitating further research.

## Results

### Characteristics of ReFRAME compounds

**Fig. 1:**
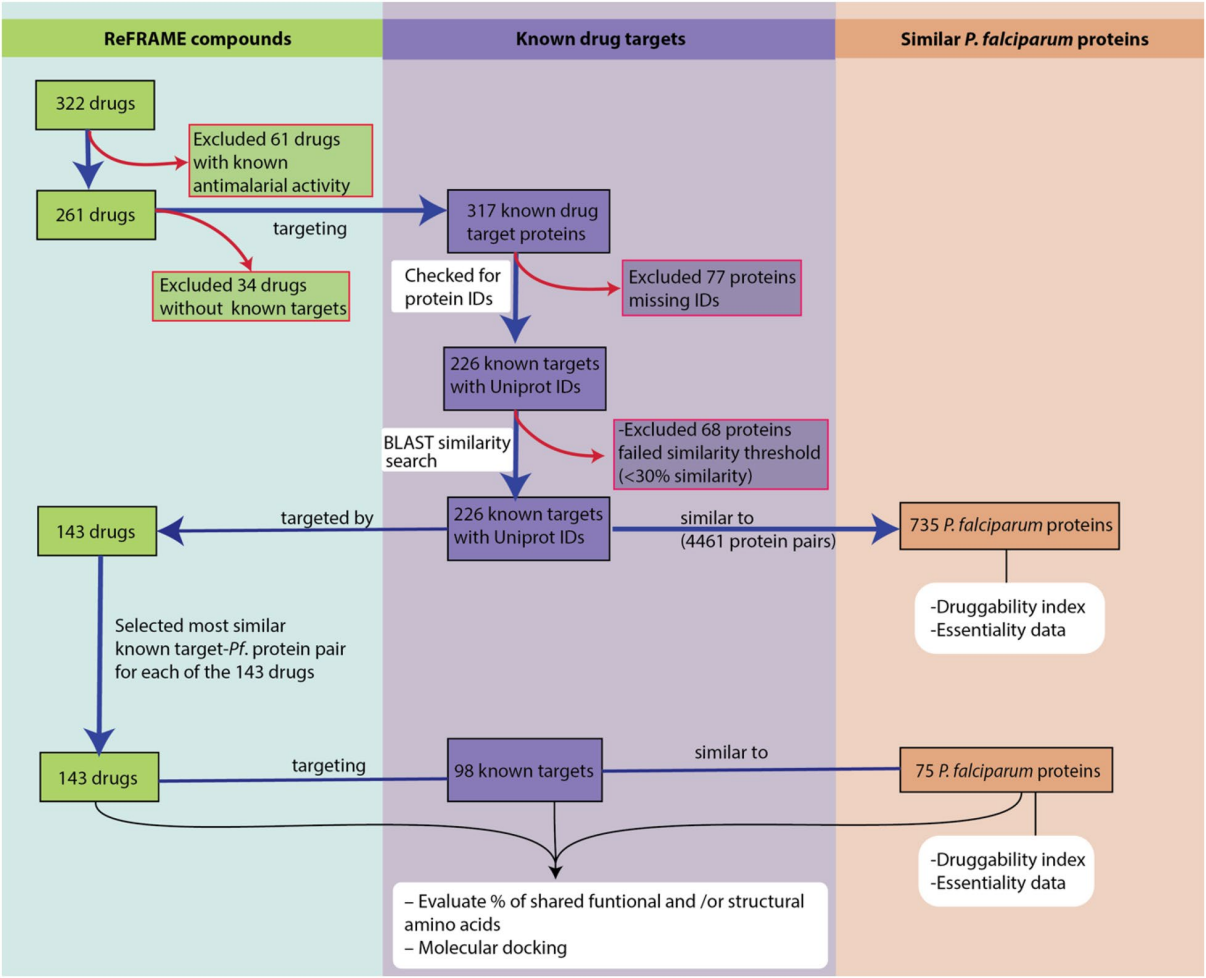
Summary of target-similarity workflow and corresponding findings. Compounds are indicated in green boxes, known drugs in purple, and predicted P*. falciparum* targets in brown

In this study, we included a total of 322 ReFRAME compounds with antiplasmodial activity on *P. falciparum* 3D7. The in vitro activity and cytotoxicity data for the ReFRAME compounds are available at https://reframedb.org/. We excluded 61 compounds from the analyses that were under investigation or already in use as antimalarials (Fig. 1). We identified at least one known protein target for 143 compounds (a total of 240 known protein targets) (Supplementary Table 1). The similarity bitscore values between these predicted targets and known targets ranged from 25 to 857. A similarity search by BLAST pairwise alignment revealed 735 *P. falciparum* proteins (predicted *P. falciparum* targets) with > 30% similarity to at least one of the 240 known targets.

### Most active compounds and their profiles

The top 10 most active compounds that we analyzed included their known and predicted target proteins (and their similarity parameters), essentiality, druggability index of the predicted *P. falciparum* target proteins, and in vitro activity of the compounds at blood and liver stages; this is shown in Table 1. Antiplasmodial drug sensitivity assays in bloodstage showed EC_50_ values ranging from 0.0006 to 9.95 μM for NVP-BGT226 and Trovafloxacin mesilate, respectively.

**Table 1:** A summary of most active ReFRAME compounds and their corresponding known and predicted target proteins

Compound	Blood stage 48 h EC_50_ (uM)	Blood stage 72 h EC_50_ (uM)	Liver 48 h EC_50_ (uM)	Known targets Uniprot ID	Avg no *Pf* of targets	Most similar *Pf* predicted target PasmoDB ID	BLAST similarity (%)*	E value*	Average bit score*	Consurf similarity %	Druggability index	Essentiality	MIS	MFS	HEK CC_50_ (uM)	HEP CC_50_ (uM)
NVP-BGT226	0.0006	0.006	0.0005	P42345	2	PF3D7_0511800	58.8%	9.17X 10^–70^	30	n/a	n/a	Essential	1	0.93	0.035	0.003
Halofuginone	0.001	0.001	0.070	P68363	5	PF3D7_0903700	61.3%	0	806	100.0%	n/a	Essential	0.599	−2.60	0.088	n/a
Quisinostat	0.001	0.003	0.008	P56524	5	PF3D7_1008000	60.1%	0	109	68.5%	0.7	Essential	0.138	−2.92	0.033	n/a
Dactinomycin	0.003	0.008	0.073	P11388	2	PF3D7_1433500	84.2%	0	857	92.3%	0.8	n/a	0.774	−2.96	0.009	0.003
Nemorubicin	0.004	0.009	0.009	P11388	2	PF3D7_1433500	83.3%	0	857	92.3%	0.8	n/a	0.774	−2.96	0.005	0.002
Bruceantin	0.005	0.007	0.000	P01106	5	PF3D7_1335400	55.2%	2.37X 10^–81^	32	54.3%	0.5	n/a	1	−2.15	0.025	n/a
Homoharringtonine	0.006	0.007	0.026	P39023	10	PF3D7_1240400	57.8%	0	28.5	n/a	n/a	n/a	1	−2.33	0.032	n/a
Cabazitaxel	0.006	0.008	0.043	P68366	5	PF3D7_0113600	55.0%	3.56X 10^–136^	28.5	n/a	n/a	Dispensable	1	−1.47	9.95	0.784
CUDC-907	0.0075	0.018	0.039	P48736	4	PF3D7_0515300	49.9%	0	133.0	72.2%	0.3	Essential	0.24	−2.67	0.027	0.003

EC_50_, half maximal effective concentration; blood stage 48 h EC_50_, the effective concentration at which 50% of the blood-stage parasites are inhibited after 48 h in culture; Blood stage 72 EC_50_, the effective concentration at which 50% of the blood-stage parasites are inhibited after 72 h in culture; Liver 48 h EC_50_ is the effective concentration at which 50% of liver-stage parasites are inhibited after 48 h in culture; Average no of targets is the average number of predicted molecular targets per known target of the compound; known targets are molecular targets that are already known for the compound; *Pf* target ID is the identifier for the predicted target in *Plasmodium falciparum*; BLAST similarity percentage (%), the percentage similarity of between the known and corresponding predicted *Pf* target based on protein-pairwise BLAST; E value, the expected number of chance alignments when comparing against a database, obtained from BLAST alignment; Bit score is a score representing the quality of sequence alignments based on BLAST; Consurf Similarity percentage % is the percentage similarity of the structural and functional amino acids (determined using the ConSurf server) between the known and predicted protein targets; Druggability index, measure of how amenable a target is to small molecule drug intervention (ranges from 0.1 (least druggable) to 1.0 (most druggable); essentiality indicates whether a gene or protein is essential for survival (essential, organism cannot survive without the protein, dispensable, organism can survive without the protein), MIS mutagenesis index score, an indicator of gene-mutability of a protein; MFS, mutagenesis fitness score, a measure of the impact of a mutation of a protein on the fitness or viability of an organism or a cell; HEK CC_50_ is the concentration which reduces number of viable human embryonic kidney cells by 50%; and HEP CC_50_ is the cytotoxic concentration which reduces number of viable hepatocytes by 50%.. A comprehensive table for all the 143 compounds is found in Supplementary Table 1

*n/a* data not available

^*^Protein similarity parameters obtained from protein BLAST pairwise alignment

### Predicted *P. falciparum* targets druggability, essentiality and docking analyses

Out of 308 predicted *P. falciparum* protein targets with druggability data, 162 (53%) proteins had druggability indices of 5 and above, suggesting moderate to high druggability (Supplementary Fig. 2). On the other hand, out of 545 predicted *P. falciparum* protein targets with essentiality data, 251 (46%) were classified as essential, while 116 (21%) were classified as dispensable (Supplementary Fig. 2). Out of 143 known–predicted target pairs, 113 (79%) shared more than 50% of functional and structural amino acids (Supplementary Table 2). The molecular docking analyses revealed that many active compounds bound to their known targets in binding pockets with binding energies that were comparable to the predicted corresponding *P. falciparum* protein targets. (Supplementary Fig. 3).

### Correlation between compound activity and similarity between known and *P.* f*alciparum *targets

**Fig. 2:**
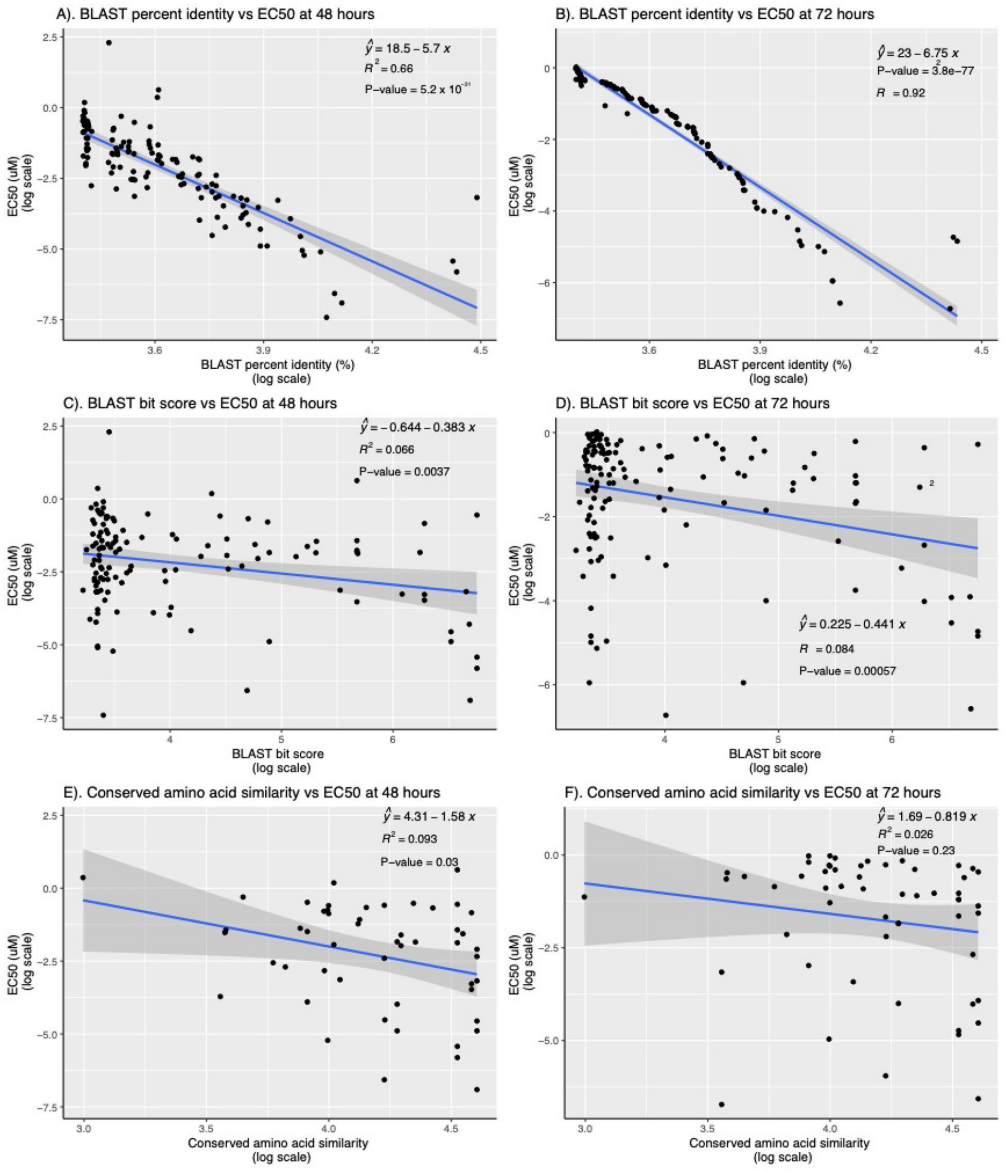
Scatter plots with fitted regression lines illustrating the association between in vitro antiplasmodial activity in 48 h and 72 h (measured as EC50 values) and BLAST metrics such as percentage identity, bit score, and the percentage of conserved amino acids. Each plot features a fitted regression line with the equation y=mx+c, indicating the statistical relationship, accompanied by shading around the line that represents the 95% confidence interval (CI). The significance of each model is denoted by the p-value, and the goodness of fit is summarized by the R_2_ value for each regression line

**Fig. 3:**
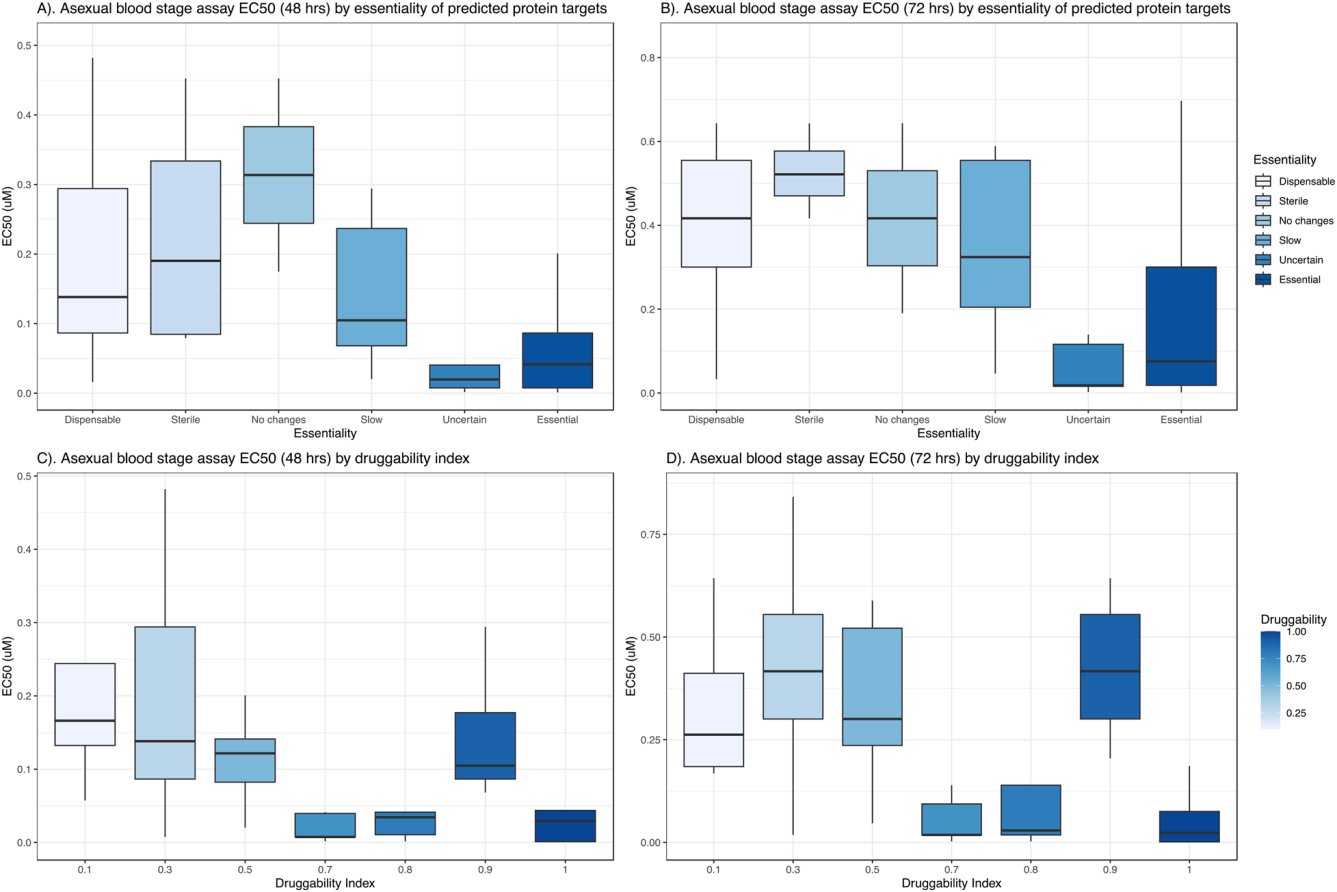
Boxplots showing the in vitro antiplasmodial activities (EC 50) of drugs predicted to target different essentiality categories of *P. falciparum* proteins (**A** and **B**) and druggability indices (**C** and **D**). Drug classifications and druggability indices were obtained from (https://tdrtargets.org/). Drugs predicted to target essential *P. falciparum* proteins, or those with uncertain effects or a druggability index of 1, exhibited the highest anti-parasitic activity. Essentiality data: slow, growth of the pathogen is slowed; sterile, organism cannot reproduce without the protein; uncertain, lack of the protein has uncertain changes; no changes, lack of the protein has no observable changes in the parasite; dispensable, organism can survive without the protein; organism cannot survive without the protein. Druggability index ranges from 0.1 (least druggable) to 1.0 (most druggable)

In vitro antiplasmodial activity (EC_50_ at 48 h) of compounds was inversely associated with the BLAST similarity bit score (beta −0.137 [standard error, SE 0.010], P value < 2.2 × 10^–16^), percentage similarity of the known–predicted target pairs (beta −0.026 [SE 0.003], P value < 2.2 × 10^–16^), and percentage of shared functional and structural amino acids between the known target-predicted protein target pairs (beta −0.059 [SE 0.007], P value < 4.6 × 10^–16^) (Table 2 and Fig. 2). These findings indicate that the compound’s in vitro antiplasmodial activity was higher with increase in similarity between its known target and predicted *P. falciparum* targets. In addition, the average number of predicted *P. falciparum* targets of a compound was positively correlated with its in vitro antiplasmodial activity (beta 0.207 [SE 0.012], P value < 2.2 × 10^–16^) (Table 2). All the observed associations were stronger in in vitro assays incubated at 72 h (Table 2). Compounds that were predicted to target *P. falciparum* proteins that were essential, uncertain, or had a druggability index of 1 had the highest in vitro antiplasmodial activity in 48- and 72-h asexual blood-stage assays (Fig. 3).

**Table 2:** Association between in vitro antiplasmodial activity of ReFRAME compounds and various factors: parameters of similarity between known–predicted protein target, average number of predicted *P. falciparum* targets and mutagenesis index score and mutagenesis fitness score of predicted *P. falciparum* targets

	Blood stage EC_50_ at 48 h		Blood stage EC_50_ at 72 h		Liver stage EC_50_ at 48 h	
	Beta (SE)	*P* value	Beta (SE)	*P* value	Beta (SE)	*P* value
BLAST percent similarity	−0.026 (0.003)	< 2.2 × 10^–16^	−0.030 (0.002)	< 2.2 × 10^–16^	−0.0060 (0.0016)	0.00011
BLAST bit score	−0.14 (0.010)	< 2.2 × 10^–16^	−0.14 (0.009)	< 2.2 × 10^–16^	−0.040 (0.0064)	6.52 × 10^–10^
Percent similarity of functional amino acids	−0.059 (0.007)	< 4.6 × 10^–16^	−0.068 (0.007)	< 2.2 × 10^–16^	−0.025 (0.0055)	8.17 × 10^–06^
Average number of predicted *P. falciparum* targets^a^	0.21 (0.012)	< 2.2 × 10^–16^	0.30 (0.011)	< 2.2 × 10^–16^	0.0022 (0.0078)	0.78
Mutagenesis index score (MIS)^b^	0.035 (0.013)	0.0054	0.041 (0.012)	0.0005	0.020 (0.0081)	0.012
Mutagenesis fitness score (MFS)^c^	0.016 (0.0072)	0.026	0.019 (0.0067)	0.0044	0.0074 (0.0046)	0.10

EC_50_, half maximal effective concentration. Univariate linear regression analyses were performed between drugs in vitro antiplasmodial activity (EC_50_ at 48 and 72 h) and percent similarity between its known targets and predicted *P. falciparum* targets (BLAST percent identity and bit score), similarity of functional and structural amino acids and number of predicted *P. falciparum* targets. EC_50_s, percentage similarity parameters and bit scores were log transformed to make them normally distributed

SE, standard error

^a^Average number of *P. falciparum* targets was determined by dividing the total number of predicted *P. falciparum* targets with the number of known targets for each drug

^b^Mutagenesis index score (MIS) indicates the comparative essentiality of *P. falciparum* genes based on the number of recovered CDS insertions relative to the potential number that could be recovered by large-scale mutagenesis [20]

^c^The Mutagenesis Fitness Score (MFS) estimates the relative growth fitness cost for mutating a gene based on its normalized quantitative insertion-site sequencing (QIseq) reads distribution [20]

## Discussion

The identification of compounds with activity against pathogens and the prioritization of those with proven activity is crucial in the processes of drug discovery and development. In this study, a target similarity in silico approach was used to predict *P. falciparum* targets for compounds that have demonstrated antiplasmodial activity, thereby prioritizing them for further development. Additionally, we discovered that the antiplasmodial activity (EC_50_) of the compounds was inversely related to the level of similarity and the percentage of shared functional and structural amino acids between the compounds' known targets and predicted *P. falciparum* protein targets. It was also positively correlated with the number of predicted *P. falciparum* protein targets, mutagenesis index score, and mutagenesis fitness score of the predicted targets. Specifically, compounds predicted to target *P. falciparum* protein targets that were classified as essential, or had a druggability index of one, exhibited higher antiplasmodial activity.

In this study, we employed a target similarity approach to predict *P. falciparum* protein targets of compounds that have demonstrated antiplasmodial activity. Understanding an antimalarial compound's target may not be essential, but is often helpful in drug discovery. For example, a compound’s structure may be modified to enhance its binding affinity to the target, thereby improving its activity [4]. Also, if the target is known, then potency can be checked on mammalian orthologues giving some indication of safety challenges that may arise without selectivity [22]. Additionally, drugs that target druggable and essential proteins in pathogens should be prioritized in drug development. Knowledge of protein targets of newly active molecules might reveal a novel mechanism of action and resistance and, ultimately, contribute to new antimalarial combination therapies. This is key in counteracting antimalarial drug resistance [10]. In our study, we conducted similarity searches on the entire *P. falciparum* proteome, a particularly advantageous approach as all the parasite’s proteins were analyzed for similarity across all life stages. It has been recommended that future antimalarial drugs target multiple life stages of the parasite's life cycle to prevent or reverse drug resistance and break the lifecycle, blocking transmission [23]. Previously, we utilized a similar approach to identify approved drugs with antiplasmodial activity [6].

We found a positive correlation between the antiplasmodial activity of compounds and the number of *P. falciparum* proteins they are predicted to target. This suggests that a compound's efficacy may increase with an increase in the number of proteins it targets, assuming the targets are validated. Compounds with multiple targets are more appealing as antimalarial drugs, as they are more likely to be potent, and pathogens are less prone to develop resistance against such molecules due to improbability of generating poly-mutations and the higher fitness cost of associated genetic changes [24, 25]. Drug-combination therapies leverage the fact that combined drugs target different pathways and possess various mechanisms of action and resistance [10]. Therefore, the positive correlation between the number of predicted *P. falciparum* targets of a compound and its antiplasmodial activity may stem from synergism resulting from the inhibition of multiple targets/pathways. Thus, the target similarity approach can complement other techniques previously employed to identify pathogen targets, such as phenotypic cellular screens [10], and in vitro drug-resistance evolution and whole genome analysis (IVIEWGA) [26].

We discovered a strong positive association between the antiplasmodial activity of the tested compounds and the similarity level between their known targets and predicted *P. falciparum* protein targets. These findings suggest that a compound's antiplasmodial activity increases with increase in similarity between its already known target and predicted *P. falciparum* protein targets. Leveraging this approach could predict the activity of various compounds against multiple organisms, as long as one of their targets is identified. This would streamline the process of repurposing compounds that have proven activity hence greatly reducing the time and resources in identifying compounds with activity. However, as our assays were cell-based, target-based functional assays are required to confirm these targets in the pathogen. Our protein-similarity approach resembles structure-based virtual screening (SBVS) and ligand-based virtual screening (LBVS) methods of predicting compound activity, which depend on in silico binding affinity or similarity to reference active compounds [27]. Both SBVS and LBVS have been employed to predict compounds with activity against *P. falciparum* [28]. The protein-similarity approach used in our study may aid in repurposing active compounds against various disease proteins or pathogens whose proteome sequences are available.

We also observed that compounds predicted to target essential *P. falciparum* proteins or those with a druggability index of 1 had the most potent antiplasmodial activity. A high druggability index implies a greater likelihood of therapeutic modulation by a small molecule if the target is essential [5]. An essential protein, crucial for pathogen survival, can be targeted to eliminate the pathogen. Hence, compounds that target *Plasmodium* proteins with high druggability and essentiality are more likely to be effective antimalarial drugs. Numerous studies have characterized *P. falciparum* targets, with data published in public databases [20, 29, 30], and there are accessible biological databases such as the TDR database (https://tdrtargets.org/) describing protein characteristics for various pathogens, including essentiality and druggability.

Several proteins predicted in our study as targets for active ReFRAME compounds are also recognized targets for established antiplasmodial agents [31]. Notably, phosphatidylinositol 3-kinase (PI3K, PF3D7_0515300), which we predicted to interact with omipalisib (EC_50_ = 0.159 μM, see Supplementary Table 1), is a validated target of artemisinins (currently the cornerstone drugs in malaria treatment) and has been linked to artemisinin resistance mechanisms [32]. Moreover, PI3K is targeted by multiple compounds in the GlaxoSmithKline library of *P. falciparum* inhibitors [33]. In the same protein family, phosphatidylinositol 4-kinase (PI4K) has been recognized as a target of imidazopyrazines, a new class of compounds with antiplasmodial activity. Imidazopyrazines inhibit the intracellular development of various *Plasmodium* species across all infection stages in the vertebrate host [34]. Notably, MMV390048, a PI4K inhibitor, exhibits potent activity against the intraerythrocytic lifecycle stages of *P. falciparum* (NF54 drug-sensitive strain), with an EC_50_ of 28 nM [35]. Our analysis also predicted the cGMP-dependent protein kinase (PKG, PF3D7_1436600) as a target of harringtonine (EC_50_ = 0.0061 μM). Screening of imidazopyridine compounds revealed PKG inhibitors with significant antiplasmodial activity, where the most potent compound (ML10) achieved an IC_50_ of 160 pM in PfPKG kinase assays and an EC_50_ of 2.1 nM against *P. falciparum* blood-stage growth in vitro [36]. Additionally, we predicted that *P. falciparum* histone deacetylase 1 (HDAC1, PF3D7_0925700) is targeted by resminostat (EC_50_ = 0.431 μM), aloxistatin (EC_50_ = 0.031 μM), and mitomycin A (EC_50_ = 0.0377 μM). This enzyme is thought to be inhibited by several compounds demonstrating substantial antimalarial activity, many with IC_50_ values below 30 nM [37]. Remarkably, a huge majority of the targets predicted in this study have not been reported in prior research, opening new avenues for developing antimalarial agents with novel mechanisms of action.

## Strengths and limitations

Our study exhibits several strengths. Firstly, we utilized a target-similarity approach to screen for potential *P. falciparum* targets of antiplasmodial compounds across the entire parasite proteome, identifying protein targets across all life stages of the parasite. Secondly, to our knowledge, this study is the first to demonstrate a correlation between the antipathogenic activity of a compound and the similarity between its known and predicted protein targets. However, the target similarity approach is only applicable to compounds with known targets, limiting the predicted targets to characterised compounds. Consequently, the diversity of the compound library directly influences the predictive outcome. For example, in this study, a significant number of compounds identified as active were anticancer agents, reflecting the ReFrame library's composition, which is enriched in anticancer drugs. To mitigate this bias and broaden the scope of potential discoveries, an additional filtering step is necessary to exclude toxic compounds either before the screening process or from the list of identified hits. Our focus has been on the asexual blood stages, the primary stage responsible for clinical malaria; the findings might not apply to other stages. Stronger binding energies in in silico molecular docking may not equate to better activity since this depends on factors like desolvation energy on binding, the binding pocket location or whether binding modulates protein function. While this study focuses on direct anti-parasitic effects, it's important to note that some compounds may exert their activity through host mechanisms, an area not investigated in this study.

## Conclusion

We employed a target similarity approach to identify potential *P. falciparum* protein targets (similar to known targets) of compounds with proven antiplasmodial activity. Future in vitro studies should validate these targets and determine their clinical relevance. We found that the antiplasmodial activity of these compounds positively correlated with the similarity between their known and predicted *P. falciparum* protein targets. Moreover, compounds targeting essential or highly druggable *P. falciparum* proteins exhibited the strongest antiplasmodial activity. Indeed, analogues of the compounds identified are often available and can be accessed from either the team who first reported the drug or through compound suppliers. This allows the rapid profiling of analogues to assess the potential for the identification of new leads with improved potency, selectivity or safety profiles. These findings suggest that the target similarity approach can be instrumental in predicting and prioritizing compounds with activity against disease proteins or pathogens. This approach may also be used to streamline and expedite drug discovery and development by repurposing compounds using information in publicly accessible biomedical databases.

### Supplementary Information

Supplementary material 1: Fig. 1. Comparative Analysis of Functional and Structural Amino Acid Similarities Between Human Tubulin Beta-1 Chain (Q9H4B7) and its Predicted *Plasmodium falciparum* Counterpart (PF3D7_1008700). Fig. 2. Histogram of showing the frequency distribution of essentiality categories (A) and druggability indices (B) of predicted *P. falciparum* (*Pf*) protein targets (A). For purposes of plotting, "embryonic lethal" and "larval arrest" categories were combined with the “Essential” categories. Fig. 3. Molecular docking and binding energies of cabazitaxel with known target P68366 (tubulin alpha-4a chain) and predicted* P. falciparum *target Q6ZLZ9 (Panel A); comparative binding affinities shown as energies (Panel B). Cabazitaxel demonstrates comparable binding affinity to the *P. falciparum* target (Q6ZLZ9), as indicated by the energy values. The conformations in Panel A represent the models with the lowest binding energies, specifically -6.9 kcal/mol and -7.1 kcal/mol. Table 1. A summary of most active ReFRAME compounds and their corresponding known and predicted target proteins. Table 2. Table showing the percentage of conserved amino acids shared between the known and predicted target pairs.

## Data Availability

The datasets generated and analyzed during the current study are available in the Similarity_Target_Prediction repository, hosted on GitHub. This repository includes all relevant data files, the R scripts used for analysis, and a codebook detailing the variables, and analytical procedures. The repository is publicly accessible and can be found at the following URL: https://github.com/rmogire/Similarity_Target_Prediction. The R scripts provided in the repository are annotated to facilitate understanding and reuse. We encourage the use and further analysis of these data in the spirit of open science and collaborative research. For any inquiries regarding the data or the methods used in this study, interested parties are encouraged to contact the corresponding authors.

## Abbreviations

*P. falciparum/Pf*: *Plasmodium falciparum*-malaria-causing parasite species
Dd2-HLH: A luciferase-expressing line of the *Plasmodium falciparum* parasite
RPMI: Roswell Park Memorial Institute cell culture medium
HEPES: 4-(2-Hydroxyethyl)-1-piperazineethanesulfonic acid
DMEM: Dulbecco's Modified Eagle Medium –a cell culture medium
DMSO: Dimethyl sulfoxide
HEK293T: Human embryonic kidney 293 cells with a temperature-sensitive SV40 T-antigen
ATCC: American Type Culture Collection-A repository of cell lines and microorganisms
PI3K: Phosphatidylinositol 3-kinase
PI4K: Phosphatidylinositol 4-kinase
PKG: cGMP-dependent protein kinase
HDAC1: Histone deacetylase 1
ReFRAME: Repurposing, Focused Rescue, and Accelerated Medchem
NCBI protein BLAST: A tool for comparing protein sequences provided by the National Center for Biotechnology Information
TDR: Tropical Disease Research
EC50: Half-maximal effective concentration
CC50: Half-maximal cytotoxic concentration
SI: Selectivity Index

## Declarations

### Disclaimer

The opinions and assertions contained herein are the private views of the authors and are not to be construed as official or as reflecting the views of the U.S. Department of the Army, the Department of Defense, or the Walter Reed Army Institute of Research.
