# Supplementary material for: Protein target similarity is positive predictor of in vitro antipathogenic activity: a drug repurposing strategy for Plasmodium falciparum
Source: J Cheminform. 2024 May 30;16:63. doi: 10.1186/s13321-024-00856-7 (PMC11145868; doi:10.1186/s13321-024-00856-7)
Supplement: Supplementary file 1 — Supplementary material 1: Fig. 1. Comparative Analysis of Functional and Structural Amino Acid Similarities Between Human Tubulin Beta-1 Chain (Q9H4B7) and its Predicted Plasmodium falciparum Counterpart (PF3D7_1008700). Fig. 2. Histogram of showing the frequency distribution of essentiality categories (A) and druggability indices (B) of predicted P. falciparum (Pf) protein targets (A). For purposes of plotting, "embryonic lethal" and "larval arrest" categories were combined with the “Essential” categories. Fig. 3. Molecular docking and binding energies of cabazitaxel with known target P68366 (tubulin alpha-4a chain) and predicted P. falciparum target Q6ZLZ9 (Panel A); comparative binding affinities shown as energies (Panel B). Cabazitaxel demonstrates comparable binding affinity to the P. falciparum target (Q6ZLZ9), as indicated by the energy values. The conformations in Panel A represent the models with the lowest binding energies, specifically -6.9 kcal/mol and -7.1 kcal/mol. Table 1. A summary of most active ReFRAME compounds and their corresponding known and predicted target proteins. Table 2. Table showing the percentage of conserved amino acids shared between the known and predicted target pairs. [file 13321_2024_856_MOESM1_ESM.docx]

**Supplementary appendix**

**Supplementary Fig. 1. Comparative Analysis of Functional and Structural Amino Acid Similarities Between Human Tubulin Beta-1 Chain (Q9H4B7) and its Predicted *Plasmodium falciparum* Counterpart (PF3D7_1008700).**

**Supplementary Fig. 2. Histogram of showing the frequency distribution of essentiality categories (A) and druggability indices (B) of predicted *P. falciparum* (*Pf*) protein targets (A). For purposes of plotting, "embryonic lethal" and "larval arrest" categories were combined with the “Essential” categories.**

**Supplementary Fig. 3: Molecular docking and binding energies of cabazitaxel with known target P68366 (tubulin alpha-4a chain) and predicted *P. falciparum* target Q6ZLZ9 (Panel A); comparative binding affinities shown as energies (Panel B).** Cabazitaxel demonstrates comparable binding affinity to the *P. falciparum* target (Q6ZLZ9), as indicated by the energy values. The conformations in Panel A represent the models with the lowest binding energies, specifically -6.9 kcal/mol and -7.1 kcal/mol.

**Supplementary Table 1. A summary of most active ReFRAME compounds and their corresponding known and predicted target proteins.**

| **Compound** | **Blood stage 48h EC_50_ (uM)** | **Blood stage 72h EC_50_ (uM)** | **Liver stage 48h EC_50_ (uM)** | **Known target** | **Predicted *P falciparum* target** | **Percentage similarity** | **E Value** | **Bit Score** | **Druggability** | **Conserved aa similarity** | **Essentiality** | **Avg no. of *Pf* target** | **Pf MIS** | **Pf MFS** | **HEK CC_50_ (uM)** | **HEP CC_50_ (uM)** |  |
| --- | --- | --- | --- | --- | --- | --- | --- | --- | --- | --- | --- | --- | --- | --- | --- | --- | --- |
| AG-024322 | n/a | 0.423 | 2.992 | P24941 | PF3D7_0102600 | 34.9 | 7.7E-64 | 34.7 | n/a | n/a | Dispensable | 39 | 1 | -0.921 | 0.145 | 0.112 |  |
| Pazinaclone | 0.077 | 0.427 | 10 | Q99928 | PF3D7_0103000 | 34.6 | 2.0E-07 | 30.8 | n/a | 43.48 | Dispensable | 2 | 0.123 | -3.123 | 9.95 | 9.95 |  |
| Pirtenidine | n/a | 0.615 | 0.838 | P00533 | PF3D7_0107600 | 33.0 | 6.8E-10 | 33.1 | n/a | n/a | Dispensable | 10 | 1 | -2.835 | 0.279 | 1.084 |  |
| Cabazitaxel | 0.006 | 0.008 | 0.043 | P68366 | PF3D7_0113600 | 55.0 | 3.6E-136 | 28.5 | n/a | n/a | Dispensable | 5 | 1 | -1.467 | 9.95 | 0.784 |  |
| 4SC-203 | n/a | 0.729 | 3.333 | P36888 | PF3D7_0203100 | 30.6 | 1.6E-41 | 52 | n/a | n/a | Dispensable | 30 | 1 | -1.603 | 3 | 4.688 |  |
| EP-475 | 0.267 | 0.313 | 10 | P07858 | PF3D7_0207700 | 36.9 | 2.8E-55 | 42.4 | n/a | n/a | Dispensable | 5 | 1 | 0.947 | 9.95 | 9.99 |  |
| Midostaurin | 0.086 | 0.417 | 3.602 | P10721 | PF3D7_0213400 | 35.7 | 3.9E-150 | 38.1 | n/a | n/a | Dispensable | 36 | 1 | 0.959 | 9.95 | n/a |  |
| TG 100572 (Hydrochloride) | 0.122 | 0.589 | 0.642 | P35968 | PF3D7_0214600 | 33.2 | 1.8E-47 | 28.9 | n/a | n/a | Dispensable | 39 | 1 | -2.581 | 1.73 | 3.437 |  |
| Doxorubicin | 0.065 | 0.25 | 1.718 | Q14978 | PF3D7_0301700 | 39.3 | 4.4E-08 | 27.7 | n/a | n/a | Dispensable | 2 | 0.898 | -2.273 | 0.074 | 0.044 |  |
| Abexinostat | 0.051 | 0.091 | 1.192 | Q13547 | PF3D7_0309800 | 42.9 | 1.0E-06 | 28.1 | n/a | n/a | Dispensable | 9 | 0.281 | -2.656 | 0.376 | n/a |  |
| CPG-52364 | 0.1 | 0.381 | 1.098 | P05129 | PF3D7_0310100 | 35.9 | 1.3E-38 | 104 | n/a | n/a | Dispensable | 32 | 1 | -3.018 | 1.638 | n/a |  |
| Lestaurtinib | 0.088 | 0.259 | 1.025 | P36888 | PF3D7_0310100 | 39.1 | 9.0E-110 | 57.4 | n/a | n/a | Dispensable | 36 | 1 | -3.018 | 0.551 | 0.029 |  |
| MX-68 | 0.175 | 0.19 | 10 | P00374 | PF3D7_0313400 | 40.6 | 3.9E-02 | 25.8 | n/a | n/a | Dispensable | 5 | 0.155 | -2.815 | 0.06 | 0.022 |  |
| LY-2874455 | 0.512 | 0.733 | 2.355 | P21802 | PF3D7_0317200 | 30.5 | 2.7E-23 | 30.8 | n/a | n/a | Dispensable | 13 | 0.325 | -2.565 | 0.875 | n/a |  |
| Leuco methylthioninium | 0.096 | 0.209 | 3.923 | P10636 | PF3D7_0318200 | 39.5 | 2.5E-01 | 29.3 | n/a | 100 | Dispensable | 1 | 0.144 | -2.838 | 3.49 | n/a |  |
| Pon/atinib | 0.294 | 0.555 | 0.241 | P11362 | PF3D7_0415300 | 33.6 | 1.5E-96 | 55.8 | 0.3 | 61.54 | Dispensable | 19 | 0.138 | -2.825 | 1.398 | 0.2 |  |
| Rimiducid | 0.059 | 0.411 | 10 | P42345 | PF3D7_0419900 | 35.8 | 3.8E-19 | 52.4 | n/a | 53.62 | n/a | 2 | 0.12 | -3.011 | 9.95 | n/a |  |
| Vindesine | 0.014 | 0.02 | 0.837 | Q71U36 | PF3D7_0422300 | 49.0 | 0 | 800 | n/a | n/a | n/a | 6 | 0.966 | -1.974 | 9.95 | 9.99 |  |
| PF-2575799 | 0.041 | 0.139 | 5.02 | Q92769 | PF3D7_0422500 | 41.6 | 6.3E-127 | 28.1 | n/a | n/a | n/a | 5 | 0.123 | -2.951 | 9.95 | n/a |  |
| XL-228 | 0.056 | 0.621 | n/a | O14965 | PF3D7_0424500 | 32.9 | 3.3E-47 | 35.4 | n/a | n/a | n/a | 32 | 1 | 0.533 | 0.537 | n/a |  |
| Protriptyline hydrochloride | 0.43 | 0.959 | 10 | P31645 | PF3D7_0425800 | 30.1 | 1.2E-07 | 27.7 | n/a | n/a | Essential | 2 | 1 | -1.972 | 9.95 | n/a |  |
| SN-38 | 0.507 | 0.358 | 1.008 | P11387 | PF3D7_0510500 | 36.1 | 3.7E+00 | 110 | 1 | 83.5 | Essential | 3 | 0.12 | -3.151 | 0.079 | n/a |  |
| XEN-723 | 0.158 | 0.334 | 10 | P13516 | PF3D7_0511200 | 36.8 | 4.5E-02 | 202 | 0.1 | 77.78 | Essential | 2 | 1 | -1.018 | 0.057 | n/a |  |
| Sapanisertib | 0.187 | 0.305 | 0.665 | P42345 | PF3D7_0511800 | 37.0 | 1.0E-03 | 30 | n/a | n/a | Essential | 2 | 1 | 0.93 | 0.102 | n/a |  |
| NVP-BGT226 | 0.001 | 0.006 | 0.001 | P42345 | PF3D7_0511800 | 58.8 | 9.2E-70 | 30 | n/a | n/a | Essential | 2 | 1 | 0.93 | 0.035 | 0.003 |  |
| Omipalisib | 0.159 | 0.158 | 0.23 | P48736 | PF3D7_0515300 | 41.4 | 1.8E-122 | 133 | 0.3 | 72.17 | Essential | 6 | 0.242 | -2.674 | 0.046 | n/a |  |
| Panulisib | 0.019 | 0.159 | 0.077 | P78527 | PF3D7_0515300 | 41.4 | 4.6E-01 | 54.3 | 0.3 | 72.17 | Essential | 4 | 0.242 | -2.674 | 0.011 | 0.01 |  |
| CUDC-907 | 0.008 | 0.018 | 0.039 | P48736 | PF3D7_0515300 | 49.9 | 0 | 133 | 0.3 | 72.17 | Essential | 4 | 0.242 | -2.674 | 0.027 | 0.003 |  |
| Oligomycin A | 0.057 | 0.168 | 0.578 | Q16665 | PF3D7_0522400 | 41.2 | 1.2E-120 | 28.5 | n/a | n/a | Essential | 3 | 1 | -2.985 | 5.579 | 0.002 |  |
| Minocycline | n/a | n/a | n/a | Q15759 | PF3D7_0615500 | 31.6 | 1.1E-79 | 63.2 | 0.5 | 77.14 | Essential | 24 | 0.133 | -2.786 | n/a | n/a |  |
| MK-1775 | 0.595 | 0.681 | 6.096 | P30291 | PF3D7_0615500 | 30.4 | 5.6E-20 | 44.7 | 0.5 | 77.14 | Essential | 16 | 0.133 | -2.786 | 0.31 | n/a |  |
| Acolbifene | 0.218 | 0.728 | 2.826 | P03372 | PF3D7_0615800 | 30.3 | 3.3E-02 | 28.1 | n/a | n/a | Essential | 2 | 0.125 | -2.915 | 2.99 | 6.206 |  |
| CE-245677 | 0.619 | 0.81 | 3.333 | P04629 | PF3D7_0628200 | 30.4 | 1.8E-08 | 28.9 | n/a | n/a | Essential | 8 | 0.14 | -2.881 | 9.95 | n/a |  |
| Echinomycin | n/a | 0.003 | 0.002 | Q9NWT6 | PF3D7_0700500 | 60.1 | 0 | 28.1 | n/a | n/a | Essential | 7 | 1 | 1.591 | 0.001 | 0.001 |  |
| CP-376395 | 0.835 | 0.884 | 6.261 | P34998 | PF3D7_0704000 | 30.1 | 1.1E-08 | 28.1 | n/a | n/a | Essential | 2 | 1 | -2.475 | 9.95 | 2.5 |  |
| Elesclomol | 0.196 | 0.437 | 10 | P17066 | PF3D7_0708800 | 34.5 | 3.6E-43 | 187 | n/a | n/a | Essential | 8 | 0.14 | -2.848 | 0.169 | n/a |  |
| Ciclesonide | 0.199 | 0.661 | 3.333 | P08185 | PF3D7_0717100 | 32.5 | 3.0E-03 | 27.3 | n/a | n/a | Essential | 3 | 1 | 0.88 | n/a | 9.95 |  |
| Darifen/Acin Hydrobromide | 1.434 | 0.324 | 10 | P20309 | PF3D7_0725300 | 36.8 | 1.2E+00 | 28.5 | n/a | 20 | Essential | 1 | 0.223 | -2.714 | 9.95 | 9.99 |  |
| CRA-026440 | 0.015 | 0.063 | 2.494 | P17655 | PF3D7_0727900 | 44.4 | 4.6E-02 | 28.1 | n/a | n/a | Essential | 5 | 1 | -0.726 | 0.836 | 0.107 |  |
| Vincristine | n/a | n/a | n/a | P07437 | PF3D7_0803700 | 54.1 | 5.0E-120 | 292 | n/a | n/a | Essential | 6 | 0.142 | -2.886 | n/a | n/a |  |
| Vinblastine sulfate | 0.029 | 0.024 | 1.104 | P07437 | PF3D7_0803700 | 48.7 | 8.7E-64 | 292 | n/a | n/a | Essential | 4 | 0.142 | -2.886 | 0.102 | n/a |  |
| Desoxyepothilone B | 0.044 | 0.076 | 0.986 | P68363 | PF3D7_0803700 | 43.7 | 5.9E-78 | 251 | n/a | n/a | Essential | 6 | 0.142 | -2.886 | 9.95 | 5.869 |  |
| Paclitaxel | 0.164 | 0.186 | 7.528 | P07437 | PF3D7_0803700 | 41.2 | 1.2E-120 | 292 | n/a | n/a | Essential | 5 | 0.142 | -2.886 | 9.95 | 7.043 |  |
| Vinorelbine | 0.171 | 0.809 | n/a | P07437 | PF3D7_0803700 | 30.4 | 7.3E-25 | 292 | n/a | n/a | Essential | 5 | 0.142 | -2.886 | 2.985 | n/a |  |
| Bazedoxifene Acetate | 0.192 | 0.636 | 5.558 | Q92731 | PF3D7_0804900 | 32.6 | 3.4E-95 | 29.6 | n/a | n/a | Essential | 2 | 0.989 | -2.411 | 3.04 | n/a |  |
| Cycloheximide | 0.178 | 0.301 | 0.109 | P49841 | PF3D7_0805700 | 37.1 | 3.0E-71 | 34.7 | n/a | n/a | Essential | 43 | 0.998 | -2.681 | 0.759 | n/a |  |
| Imipramine hydrochloride | 0.18 | 0.963 | 10 | P08173 | PF3D7_0819200 | 30.0 | 2.0E-62 | 28.9 | n/a | n/a | Essential | 3 | 0.141 | -2.919 | 9.95 | 9.95 |  |
| Propafenone SR | 0.253 | 0.566 | 10 | Q8NHY2 | PF3D7_0822800 | 33.3 | 2.9E-45 | 57.8 | n/a | 48.58 | Essential | 32 | 0.738 | -2.605 | 9.95 | 9.99 |  |
| Golvatinib | 0.738 | 1.016 | 2.836 | P35968 | PF3D7_0902100 | 30.0 | 7.0E-16 | 30 | n/a | n/a | Essential | 39 | 1 | -2.697 | 4.363 | n/a |  |
| INO-5042 | n/a | 0.403 | 2.066 | P04637 | PF3D7_0902600 | 35.8 | 1.0E+01 | 27.7 | n/a | 62.5 | Essential | 1 | n/a | n/a | 1.331 | 0.705 |  |
| Halofuginone | 0.001 | 0.001 | 0.07 | P68363 | PF3D7_0903700 | 61.3 | 0 | 806 | n/a | 100 | Essential | 5 | 0.599 | -2.602 | 0.088 | n/a |  |
| Gentian violet | 0.104 | 0.456 | 0.214 | P19438 | PF3D7_0904300 | 34.5 | 1.5E-04 | 27.3 | n/a | n/a | n/a | 6 | 1 | -0.416 | 0.136 | n/a |  |
| GV 150013 | n/a | 0.253 | 10 | P32239 | PF3D7_0905700 | 39.3 | 1.7E+00 | 29.6 | n/a | n/a | n/a | 3 | n/a | n/a | 9.95 | n/a |  |
| Bortezomib | 0.091 | 0.082 | 0.012 | P20618 | PF3D7_0908800 | 43.3 | 1.0E-03 | 29.6 | n/a | n/a | n/a | 4 | 0.123 | -3.009 | 0.025 | n/a |  |
| TMC-353121 | 0.319 | 0.65 | 10 | P03420 | PF3D7_0916300 | 32.5 | 3.4E-28 | 29.3 | n/a | n/a | n/a | 4 | 0.138 | -3.23 | 9.95 | n/a |  |
| SGI-1776 | 0.482 | 0.623 | 1.793 | Q9P1W9 | PF3D7_0918300 | 32.6 | 4.8E-95 | 27.3 | n/a | n/a | n/a | 22 | 0.123 | -2.983 | 4.575 | 1.038 |  |
| Atiprimod dimaleate | 0.043 | 0.43 | 7.331 | P40763 | PF3D7_0918400 | 34.6 | 2.8E-50 | 29.3 | n/a | 57.14 | n/a | 1 | 1 | -0.536 | 1.715 | 2.989 |  |
| Aloxistatin | 0.031 | 0.069 | 10 | Q13547 | PF3D7_0925700 | 44.2 | 1.3E-129 | 535 | 0.8 | 97.8 | n/a | 5 | 0.128 | -2.762 | 9.95 | n/a |  |
| Mitomycin A | 0.038 | 0.018 | 1.36 | Q13547 | PF3D7_0925700 | 51.4 | 0 | 535 | 0.8 | 97.8 | n/a | 9 | 0.128 | -2.762 | 0.362 | 0.032 |  |
| Resminostat | 0.431 | 0.697 | 7.058 | Q13547 | PF3D7_0925700 | 30.7 | 8.2E-44 | 535 | 0.8 | 97.8 | n/a | 3 | 0.128 | -2.762 | 3.551 | 1.196 |  |
| CUDC-101 | 0.041 | 0.052 | 0.349 | P04626 | PF3D7_0926000 | 46.4 | 1.2E-02 | 31.2 | n/a | n/a | Essential | 9 | 1 | -1.657 | 0.408 | n/a |  |
| Trametinib dimethyl sulfoxide | 0.16 | 0.3 | n/a | P36507 | PF3D7_0926000 | 38.4 | 1.0E-172 | 33.5 | n/a | n/a | Essential | 40 | 1 | -1.657 | 9.95 | 0.119 |  |
| Dacomitinib | 0.54 | 0.953 | 0.596 | P00533 | PF3D7_0926000 | 30.1 | 1.3E-38 | 31.2 | n/a | n/a | Essential | 10 | 1 | -1.657 | 2.624 | n/a |  |
| HKI-357 | 0.212 | 0.619 | 0.573 | P04626 | PF3D7_0926000 | 33.0 | 2.0E-56 | 31.2 | n/a | n/a | Essential | 10 | 1 | -1.657 | 1.724 | n/a |  |
| Valspodar | 0.307 | 0.37 | 0.003 | P00742 | PF3D7_0930300 | 36.0 | 5.8E-13 | 31.2 | n/a | n/a | Dispensable | 10 | 0.141 | -3.197 | 9.95 | n/a |  |
| Quisinostat | 0.001 | 0.003 | 0.008 | P56524 | PF3D7_1008000 | 60.1 | 0 | 109 | 0.7 | 68.49 | Essential | 5 | 0.138 | -2.924 | 0.033 | n/a |  |
| Ricolinostat | 0.555 | 0.77 | 4.374 | Q9UBN7 | PF3D7_1008000 | 30.4 | 1.6E-13 | 85.5 | 0.7 | 68.49 | Essential | 2 | 0.138 | -2.924 | 9.95 | n/a |  |
| Belinostat | 0.09 | 0.188 | 1.828 | Q969S8 | PF3D7_1008000 | 41.2 | 4.1E-126 | 92 | 0.7 | 68.49 | Essential | 5 | 0.138 | -2.924 | 1.289 | n/a |  |
| Acranil | 0.008 | 0.02 | 0.312 | Q9H4B7 | PF3D7_1008700 | 49.0 | 0 | 679 | 1 | 100 | Essential | 4 | 0.305 | -2.901 | 1.433 | 1.11 |  |
| Docetaxel | 0.011 | 0.011 | 0.401 | Q9H4B7 | PF3D7_1008700 | 54.7 | 7.1E-81 | 679 | 1 | 100 | Essential | 4 | 0.305 | -2.901 | 9.95 | 0.024 |  |
| Ixabepilone | 0.042 | n/a | 0.867 | Q13509 | PF3D7_1008700 | 89.1 | 0 | 778 | 1 | 100 | Essential | 7 | 0.305 | -2.901 | 0.015 | 0.007 |  |
| Birin/apant | 0.19 | 0.603 | 10 | Q13490 | PF3D7_1013000 | 33.0 | 6.1E-47 | 32 | n/a | n/a | Essential | 11 | 0.713 | -0.407 | 9.95 | n/a |  |
| Astemizole | 0.342 | 0.853 | 0.05 | O95259 | PF3D7_1017000 | 30.3 | 9.7E-18 | 30.8 | n/a | n/a | Essential | 2 | 0.128 | -3.097 | 9.95 | n/a |  |
| Chromomycin A3 | 0.031 | 0.048 | 0.03 | Q13547 | PF3D7_1018200 | 46.7 | 6.3E-17 | 30.4 | n/a | n/a | Essential | 9 | 0.243 | -2.701 | 0.051 | 0.055 |  |
| Batimastat | 0.095 | 0.165 | 10 | P39900 | PF3D7_1018900 | 41.3 | 1.8E-121 | 31.6 | n/a | n/a | Essential | 3 | 0.237 | -2.728 | 9.95 | n/a |  |
| Bardoxolone methyl | 0.34 | 0.75 | 0.155 | P25963 | PF3D7_1021900 | 30.4 | 2.0E-23 | 35.8 | n/a | 61.9 | Essential | 13 | 0.385 | -2.603 | 0.208 | 0.557 |  |
| Darapladib | 0.515 | 0.85 | 2.876 | P47712 | PF3D7_1038800 | 30.3 | 2.1E-17 | 32.7 | 0.5 | 63.64 | Essential | 2 | 1 | -2.763 | 1.733 | n/a |  |
| Ridinilazole | n/a | 0.09 | 0.215 | Q13547 | PF3D7_1112300 | 43.2 | 2.7E-77 | 29.3 | n/a | n/a | Slow | 9 | 0.128 | -3.134 | 9.95 | 3.109 |  |
| Flumatinib mesylate | 0.085 | 0.213 | 10 | P00519 | PF3D7_1121300 | 39.4 | 5.1E-12 | 52 | n/a | n/a | Slow | 19 | 1 | -0.436 | 9.95 | n/a |  |
| Barasertib-HQPA | 0.253 | 0.539 | 4.618 | Q96GD4 | PF3D7_1121900 | 34.0 | 2.3E-44 | 90.9 | n/a | n/a | Slow | 34 | 0.249 | -3.434 | 9.95 | 9.99 |  |
| Tryptanthrin | 0.737 | 0.562 | 0.041 | P14902 | PF3D7_1129600 | 33.3 | 5.8E+00 | 26.9 | n/a | 38.46 | Slow | 1 | 1 | -2.223 | 9.95 | n/a |  |
| Desipramine | 0.238 | 0.622 | 3.333 | P31645 | PF3D7_1132500 | 32.9 | 2.1E-95 | 33.5 | 0.5 | 35.85 | Slow | 3 | 1 | -0.049 | 9.95 | n/a |  |
| Alectinib Hydrochloride | 0.907 | 0.926 | 0.152 | Q9UM73 | PF3D7_1136500 | 30.1 | 1.2E-07 | 30 | n/a | n/a | Slow | 8 | n/a | n/a | 0.797 | n/a |  |
| Chlorhexidine Dressing | 0.011 | 0.111 | 2.536 | P33402 | PF3D7_1138400 | 42.9 | 2.7E+00 | 65.9 | n/a | 68.64 | Slow | 1 | 0.954 | -2.882 | 2.208 | 0.65 |  |
| Lerisetron | 0.144 | 0.267 | 1.134 | P46098 | PF3D7_1140500 | 38.9 | 6.0E-02 | 28.9 | n/a | n/a | Slow | 5 | 1 | -2.507 | 9.95 | 0.236 |  |
| Puromycin | 0.061 | 0.125 | 0.84 | Q9UNX3 | PF3D7_1142800 | 42.2 | 1.2E-122 | 27.7 | n/a | n/a | Slow | 6 | 0.171 | -2.815 | 1.245 | 0.498 |  |
| Methylthioninium Chloride | 0.067 | 0.118 | 1.054 | P29475 | PF3D7_1143100 | 42.9 | 1.6E-10 | 29.3 | n/a | 45.71 | Slow | 1 | 0.406 | -2.557 | 1.774 | n/a |  |
| Brigatinib | 0.099 | 0.346 | 3.333 | P00533 | PF3D7_1145200 | 32.5 | 5.7E-79 | 38.5 | n/a | n/a | Slow | 14 | 1 | -2.278 | 1.386 | 0.136 |  |
| Crizotinib | 0.225 | 0.827 | 8.516 | P08581 | PF3D7_1148000 | 30.3 | 6.5E-39 | 37 | 0.5 | 50 | Slow | 10 | 0.119 | -2.973 | 9.95 | n/a |  |
| Panobinostat Lactate | 0.02 | 0.051 | 0.131 | P00533 | PF3D7_1148000 | 46.4 | 1.2E-02 | 47 | 0.5 | 50 | Slow | 10 | 0.119 | -2.973 | 0.078 | n/a |  |
| Penfluridol | 0.218 | 0.524 | 3.167 | P08684 | PF3D7_1205700 | 34.0 | 5.0E-03 | 32.7 | 0.3 | 35.71 | Slow | 2 | 1 | -1.692 | 1.901 | n/a |  |
| ZSTK-474 | 0.238 | 0.841 | 0.973 | P48736 | PF3D7_1212300 | 30.3 | 4.1E-36 | 29.6 | n/a | n/a | Slow | 6 | 0.999 | -1.224 | 0.762 | n/a |  |
| Trovafloxacin mesilate | 9.95 | 0.669 | 10 | P43700 | PF3D7_1219000 | 32.2 | 1.2E-78 | 31.6 | n/a | n/a | Slow | 3 | 1 | -2.285 | 9.95 | n/a |  |
| Epirubicin HCl | 0.271 | 0.366 | 1.484 | O14646 | PF3D7_1220900 | 36.0 | 2.3E-142 | 33.5 | n/a | n/a | Slow | 9 | 0.129 | -3.236 | 0.103 | n/a |  |
| Alvespimycin hydrochloride | 0.159 | 0.273 | 0.129 | P07900 | PF3D7_1222300 | 38.8 | 1.2E-73 | 514 | n/a | n/a | Slow | 9 | 0.148 | -2.824 | 0.164 | n/a |  |
| Aminoquinuride | 0.079 | 0.449 | 3.494 | P15917 | PF3D7_1228300 | 34.5 | 4.6E-46 | 28.1 | n/a | n/a | Slow | 6 | 0.806 | -2.349 | 9.95 | 9.99 |  |
| Sepantronium bromide | 0.021 | 0.082 | 0.01 | P28074 | PF3D7_1230400 | 43.5 | 4.6E-04 | 33.9 | n/a | n/a | Slow | 5 | 0.431 | -2.873 | 0.056 | n/a |  |
| Homoharringtonine | 0.006 | 0.007 | 0.026 | P39023 | PF3D7_1240400 | 57.8 | 0 | 28.5 | n/a | n/a | n/a | 10 | 1 | -2.332 | 0.032 | n/a |  |
| Procyclidine hydrochloride | 0.594 | 0.437 | 10 | P11229 | PF3D7_1240600 | 34.6 | 6.4E-02 | 29.3 | n/a | n/a | n/a | 3 | 1 | -1.848 | 9.95 | n/a |  |
| TG-100801 | 0.068 | 0.205 | 1.408 | P35916 | PF3D7_1247500 | 39.7 | 1.7E-106 | 33.5 | n/a | n/a | n/a | 31 | 1 | -0.636 | 9.95 | n/a |  |
| CB-5083 | 0.233 | 0.608 | 0.54 | P55072 | PF3D7_1311500 | 30.4 | 6.5E-51 | 203 | n/a | n/a | n/a | 35 | 0.924 | -2.109 | 0.469 | 0.468 |  |
| Foretinib | 0.139 | 0.859 | 6.05 | P35968 | PF3D7_1315100 | 30.2 | 1.3E-63 | 72 | n/a | 73.33 | n/a | 21 | 0.12 | -3.052 | 2.369 | n/a |  |
| XL-888 | 0.201 | 0.348 | 0.099 | O60674 | PF3D7_1315100 | 36.3 | 1.4E-51 | 76.6 | n/a | 73.33 | n/a | 17 | 0.12 | -3.052 | 0.047 | n/a |  |
| Lomitapide | 0.277 | 0.82 | 2.254 | P55157 | PF3D7_1316900 | 30.3 | 8.8E-05 | 28.9 | n/a | n/a | n/a | 2 | 0.128 | -3.034 | 0.791 | n/a |  |
| Indacaterol maleate | 0.682 | 0.951 | 3.114 | P07550 | PF3D7_1319300 | 30.1 | 1.2E-07 | 28.5 | n/a | n/a | n/a | 3 | 0.122 | -3.02 | 9.95 | n/a |  |
| CP 66948 | 0.615 | 0.977 | 10 | P25021 | PF3D7_1319900 | 30.0 | 3.0E-03 | 29.6 | n/a | 50 | n/a | 2 | 1 | -2.315 | 9.95 | 9.95 |  |
| Bruceantin | 0.005 | 0.007 | 0 | P01106 | PF3D7_1335400 | 55.3 | 2.4E-81 | 32.7 | 0.5 | 54.35 | n/a | 5 | 1 | -2.153 | 0.025 | n/a |  |
| RTA-63415 | 0.471 | 0.761 | 0.307 | Q16236 | PF3D7_1335400 | 30.4 | 1.2E-35 | 33.1 | 0.5 | 54.35 | n/a | 5 | 1 | -2.153 | 0.325 | n/a |  |
| AT-9283 | 0.129 | 0.864 | 0.569 | O14965 | PF3D7_1337100 | 30.2 | 8.9E-45 | 120 | n/a | n/a | n/a | 30 | 0.127 | -2.795 | 4.792 | 1.339 |  |
| RGB-286638 | 0.138 | 0.3 | 0.087 | P50750 | PF3D7_1337100 | 37.3 | 8.3E-74 | 169 | n/a | n/a | n/a | 36 | 0.127 | -2.795 | 0.058 | n/a |  |
| Mibefradil dihydrochloride | n/a | n/a | n/a | Q13936 | PF3D7_1341600 | 30.8 | 1.2E-27 | 30 | n/a | n/a | n/a | 3 | 0.488 | -2.397 | n/a | n/a |  |
| MG-516 | 0.144 | 0.673 | 2.178 | P07949 | PF3D7_1349300 | 32.2 | 2.9E-91 | 90.9 | 0.5 | 55.73 | n/a | 25 | 0.927 | -2.909 | 2.248 | 1.457 |  |
| Tesevatinib | n/a | n/a | n/a | P54760 | PF3D7_1349300 | 32.5 | 3.8E-43 | 74.3 | 0.5 | 55.73 | n/a | 9 | 0.927 | -2.909 | n/a | n/a |  |
| AC-430 | 1.199 | 0.924 | 4.211 | O60674 | PF3D7_1349300 | 30.1 | 2.1E-32 | 79.3 | 0.5 | 55.73 | n/a | 27 | 0.927 | -2.909 | 9.95 | n/a |  |
| Becatecarin | n/a | 0.194 | 7.16 | P11388 | PF3D7_1352800 | 40.6 | 1.4E-124 | 32.3 | n/a | n/a | n/a | 2 | 0.122 | -3.008 | 0.143 | 0.158 |  |
| NCO 700 | 0.016 | 0.033 | 10 | P07858 | PF3D7_1353300 | 47.4 | 1.2E-01 | 26.6 | n/a | n/a | n/a | 5 | 1 | -1.963 | 9.95 | 9.95 |  |
| Hydroxyprogesterone caproate | 0.55 | 0.742 | 1.71 | P06401 | PF3D7_1356000 | 30.4 | 2.0E-24 | 30.8 | n/a | 54.55 | n/a | 2 | 1 | -0.727 | 9.95 | 0.863 |  |
| BMS-983970 | 0.063 | 0.084 | 0.102 | Q14524 | PF3D7_1358600 | 43.3 | 8.2E-06 | 30 | n/a | n/a | n/a | 6 | 1 | 1.384 | 9.95 | 0.059 |  |
| TCD-717 | 0.063 | 0.714 | 0.102 | P35790 | PF3D7_1373500 | 30.7 | 3.8E-22 | 31.2 | n/a | n/a | n/a | 5 | n/a | n/a | 1.929 | 4.769 |  |
| Lapatinib | 0.3 | 0.519 | 4.215 | P00915 | PF3D7_1408900 | 34.1 | 3.2E-06 | 27.3 | n/a | n/a | n/a | 7 | 1 | -0.542 | 9.95 | n/a |  |
| Acivicin | 0.209 | 0.545 | 1.1 | P17812 | PF3D7_1410200 | 34.0 | 6.3E-20 | 117 | n/a | 94.41 | n/a | 2 | 0.143 | -2.778 | 7.727 | 1.703 |  |
| Penbutolol | 0.418 | 0.98 | 20 | P08588 | PF3D7_1410300 | 30.0 | 1.9E-07 | 30 | 0.5 | 54.55 | n/a | 1 | 1 | -2.289 | 9.95 | n/a |  |
| L 796568 | n/a | 0.277 | 3.333 | P08588 | PF3D7_1410300 | 34.5 | 3.8E-56 | 30 | 0.5 | 54.55 | n/a | 2 | 1 | -2.289 | 1.628 | 1.064 |  |
| TYROTHRICIN | n/a | 0.033 | 0.193 | P00722 | PF3D7_1411300 | 47.2 | 1.2E-04 | 34.7 | n/a | 60 | n/a | 1 | 0.229 | -3.297 | 0.263 | 2.07 |  |
| CHR-3996 | 0.02 | 0.015 | 0.151 | Q13547 | PF3D7_1423200 | 53.2 | 1.6E-78 | 28.5 | n/a | n/a | n/a | 7 | 1 | -1.097 | 0.92 | 0.195 |  |
| ZM447439 | 0.452 | 0.643 | 4.33 | O14965 | PF3D7_1423600 | 32.6 | 3.4E-95 | 131 | n/a | 53.5 | n/a | 32 | 0.936 | -0.774 | 9.95 | n/a |  |
| Anisomycin | 0.044 | 0.061 | 0.266 | P62750 | PF3D7_1429700 | 45.5 | 8.0E-177 | 25 | n/a | n/a | n/a | 7 | 0.121 | -2.989 | 0.082 | n/a |  |
| Datelliptium Chloride | n/a | 0.193 | 10 | Q02880 | PF3D7_1433500 | 40.6 | 1.4E-124 | 294 | 0.8 | 92.31 | n/a | 2 | 0.774 | -2.962 | 4.686 | 0.505 |  |
| Idarubicin | 1.874 | 0.302 | 0.843 | P11388 | PF3D7_1433500 | 37.0 | 3.7E-04 | 292 | 0.8 | 92.31 | n/a | 2 | 0.774 | -2.962 | 0.036 | n/a |  |
| Mitoxantrone | 0.239 | 0.357 | 1.722 | P11388 | PF3D7_1433500 | 36.1 | 4.1E-13 | 292 | 0.8 | 92.31 | n/a | 2 | 0.774 | -2.962 | 0.217 | n/a |  |
| Dactinomycin | 0.003 | 0.008 | 0.073 | P11388 | PF3D7_1433500 | 84.2 | 0 | 857 | 0.8 | 92.31 | n/a | 2 | 0.774 | -2.962 | 0.009 | 0.003 |  |
| Nemorubicin | 0.004 | 0.009 | 0.009 | P11388 | PF3D7_1433500 | 83.3 | 0 | 857 | 0.8 | 92.31 | n/a | 2 | 0.774 | -2.962 | 0.005 | 0.002 |  |
| Aclarubicin | 0.154 | 0.302 | 2.226 | Q02880 | PF3D7_1433500 | 37.0 | 3.1E-02 | 294 | 0.8 | 92.31 | n/a | 2 | 0.774 | -2.962 | 0.166 | n/a |  |
| TAS-103 | 0.574 | 0.756 | 1.499 | P11388 | PF3D7_1433500 | 30.4 | 3.4E-08 | 857 | 0.8 | 92.31 | n/a | 2 | 0.774 | -2.962 | 0.334 | 0.766 |  |
| UCN-01 | 0.079 | 0.488 | 2.147 | Q96LZ3 | PF3D7_1434200 | 34.3 | 2.2E-66 | 37 | n/a | n/a | n/a | 23 | 0.133 | -3.037 | 0.074 | n/a |  |
| Quinidine | 0.041 | 0.084 | 10 | O00180 | PF3D7_1436300 | 43.3 | 1.1E-55 | 28.5 | n/a | n/a | n/a | 7 | 1 | -2.182 | 9.95 | n/a |  |
| Halofantrine HCl | n/a | 0.001 | 1.883 | Q12809 | PF3D7_1436600 | 82.6 | 0 | 55.1 | n/a | 35.04 | n/a | 2 | 0.179 | -2.721 | 9.95 | 9.95 |  |
| Harringtonine | 0.024 | 0.043 | 0.029 | Q12809 | PF3D7_1436600 | 47.1 | 1.1E+00 | 55.1 | n/a | 35.04 | n/a | 2 | 0.179 | -2.721 | 0.185 | 1.708 |  |
| Acetrapib | 0.63 | 0.72 | 10 | P11597 | PF3D7_1440100 | 30.0 | 5.8E+00 | 29.3 | n/a | n/a | n/a | n/a | 0.341 | -2.607 | 9.95 | 9.99 |  |
| Luminespib | 0.038 | 0.04 | 0.029 | P46598 | PF3D7_1443900 | 47.2 | 1.5E-09 | 438 | n/a | n/a | n/a | 6 | 0.135 | -3.033 | 0.011 | n/a |  |
| Voclosporin | n/a | 0.254 | 0 | P63098 | PF3D7_1451700 | 39.2 | 8.8E-114 | 168 | 0.9 | 100 | n/a | 18 | 0.996 | 0 | 9.95 | 9.95 |  |
| JTC-801 | 0.123 | 0.635 | 0.429 | P41146 | PF3D7_1457400 | 32.6 | 1.3E-06 | 27.7 | 0.5 | 100 | n/a | 2 | 0.141 | -2.874 | 1.81 | N/A |  |
| Desmethylastemizole | 0.023 | 0.047 | 0.07 | P08047 | PF3D7_1473900 | 46.7 | 6.3E-17 | 28.5 | n/a | n/a | n/a | 5 | 0.999 | -2.129 | 5.919 | 0.058 |  |
| *Protein similarity parameters obtained from protein BLAST pairwise alignment. Abbreviations: n/a , data not available; EC_50_, half maximal effective concentration; blood stage 48h EC_50_, the effective concentration at which 50% of the blood-stage parasites are inhibited after 48 hours in culture; Blood stage 72 EC_50_, the effective concentration at which 50% of the blood-stage parasites are inhibited after 72 hours in culture; Liver 48h EC_50_ is the effective concentration at which 50% of liver-stage parasites are inhibited after 48 hours in culture; Average no of targets is the average number of predicted molecular targets per known target of the compound; known targets are molecular targets that are already known for the compound; *Pf* target ID is the identifier for the predicted target in *Plasmodium falciparum*; BLAST similarity percentage (%), the percentage similarity of between the known and corresponding predicted *Pf* target based on protein-pairwise BLAST; E value, the expected number of chance alignments when comparing against a database, obtained from BLAST alignment; Bit score is a score representing the quality of sequence alignments based on BLAST; Consurf Similarity percentage % is the percentage similarity of the structural and functional amino acids (determined using the ConSurf server ) between the known and predicted protein targets ; Druggability index, measure of how amenable a target is to small molecule drug intervention (ranges from 0.1 (least druggable) to 1.0 (most druggable); essentiality indicates whether a gene or protein is essential for survival (essential, organism cannot survive without the protein, dispensable, organism can survive without the protein), MIS mutagenesis index score, an indicator of gene-mutability of a protein; MFS, mutagenesis fitness score, a measure of the impact of a mutation of a protein on the fitness or viability of an organism or a cell; HEK CC_50_ is the concentration which reduces number of viable human embryonic kidney cells by 50%; and HEP CC_50_ is the cytotoxic concentration which reduces number of viable hepatocytes by 50%. | | | | | | | | | | | | | | | | | |

**Supplementary Table 2: Table showing the percentage of conserved amino acids shared between the known and predicted target pairs**

| Percentage of shared conserved amino acids | Number of known – predicted target pairs |
| --- | --- |
| 90-100% | 54 |
| 70-90% | 15 |
| 50-70% | 49 |
| 30-50% | 18 |
| <30% | 5 |
